# Supplementary material for: WISDOM randomized trial comparing risk-based versus annual breast cancer screening: study cohort characteristics and design
Source: NPJ Breast Cancer. 2026 May 24;12:108. doi: 10.1038/s41523-026-00952-x (PMC13478457; doi:10.1038/s41523-026-00952-x)
Supplement: Supplementary file 1 [file 41523_2026_952_MOESM1_ESM.docx]

**SUPPLEMENTARY INFORMATION**

WISDOM Randomized Trial Comparing Risk-Based Versus Annual Breast Cancer Screening: Baseline Characteristics and Study Conduct

Fiscalini et al.

**Table of Contents**

[Supplementary Table S1: Minority enrollment in WISDOM	2](#_Toc216181712)

[Supplementary Table S2: List of clinical sites	3](#_Toc216181713)

[Supplementary Table S3: Factors affecting patient participation choices	4](#_Toc216181714)

[Supplementary Table S4. Risk assessment and screening recommendations	5](#_Toc216181715)

Supplementary Table S5: Athena Breast Health Network and WISDOM Consortia Members……….6

## Supplementary Table S1: Minority enrolment in WISDOM

Year-by-year rates of enrolment to WISDOM categorized by self-reported race/ethnicity

|  | **White, NH** | **Black or African American** | **Native Hawaiian or Other Pac Islander/American Indian or Alaska Native, NH** | **Asian, NH** | **Two or More Race, NH** | **Hispanic or Latino** | **Unknown/Pref not to answer/other** |
| --- | --- | --- | --- | --- | --- | --- | --- |
| **Start-2019** | 81.4% | 1.7% | 0.3% | 4.5% | 2.7% | 8.1% | 1.2% |
| **2020** | 74.9% | 4.1% | 0.4% | 5.9% | 3.3% | 10.4% | 1.0% |
| **2021** | 73.4% | 8.1% | 0.5% | 4.0% | 3.1% | 10.2% | 0.7% |
| **2022** | 67.4% | 11.5% | 0.6% | 4.4% | 3.9% | 11.4% | 0.7% |
| **2023** | 59.4% | 17.5% | 0.8% | 5.8% | 3.1% | 11.8% | 1.5% |
| **All Time** | 75.6% | 5.6% | 0.4% | 4.6% | 3.1% | 9.6% | 1.0% |

## Supplementary Table S2: List of clinical sites

List of WISDOM recruitment sites and years of enrolment in WISDOM 1.0

| **Site** | **Year**  **Site Opened** |
| --- | --- |
| **Initial sites** |  |
| University of California San Francisco | 2016 |
| University of California San Diego | 2016 |
| University of California Los Angeles | 2017 |
| University of California Davis | 2017 |
| University of California Irvine | 2017 |
| Sanford Health | 2017 |
| **Expansion sites** |  |
| University of Chicago | 2019 |
| University of Alabama at Birmingham | 2020 |
| Diagnostic Center of Miami | 2020 |
| Louisiana State University | 2020 |
| Veterans Health Administration* | 2021 |

*Recruitment only through select VA centers

## Supplementary Table S3: Factors affecting patient participation choices

Multivariate Analysis Comparing Participant Choice of Randomized versus Self-Selection into Annual or Personalized. NH = Non-Hispanic

|  | **Self-Select Risk-based vs Randomized** (complete cases) | | | | **Self-Select Annual vs  Randomized** (complete cases) | | | |
| --- | --- | --- | --- | --- | --- | --- | --- | --- |
|  | **RR** | **Lower CI: 2.5%** | **Upper CI: 97.5%** | **p*** | **RR** | **Lower CI: 2.5%** | **Upper CI: 97.5%** | **p*** |
| **Age (years) at Group Selected Date** | | | | | | | | |
| <50 | ref | ref | ref | ref | ref | ref | ref | ref |
| 50-59 | 0.92 | 0.88 | 0.96 | 0.0001 | 1.27 | 1.06 | 1.51 | 0.0089 |
| 60-69 | 0.87 | 0.83 | 0.92 | <0.0001 | 1.67 | 1.36 | 2.05 | <0.0001 |
| 70-79 | 0.75 | 0.69 | 0.82 | <0.0001 | 2.02 | 1.57 | 2.60 | <0.0001 |
| **Race, Ethnicity** | | | | | | | | |
| NH White | ref | ref | ref | ref | ref | ref | ref | ref |
| Hispanic | 1.18 | 1.13 | 1.23 | <0.0001 | 1.42 | 1.19 | 1.70 | 0.0001 |
| NH Asian | 1.20 | 1.13 | 1.28 | <0.0001 | 1.33 | 1.02 | 1.74 | 0.0383 |
| NH Black or African American | 1.02 | 0.96 | 1.09 | 0.5047 | 1.75 | 1.44 | 2.12 | <0.0001 |
| NH More than one race | 1.16 | 1.08 | 1.25 | 0.0001 | 0.76 | 0.51 | 1.14 | 0.1847 |
| NH Native Hawaiian or Other Pacific Islander/American Indian or Alaska Native | 0.91 | 0.72 | 1.15 | 0.4357 | 0.85 | 0.33 | 2.22 | 0.7455 |
| Other/Unknown | 1.41 | 1.25 | 1.58 | <0.0001 | 1.78 | 1.06 | 2.99 | 0.0294 |
| **Family History at Baseline** | | | | | | | | |
| No First or Second Degree Relative | ref | ref | ref | ref | ref | ref | ref | ref |
| First Degree Relative | 1.49 | 1.42 | 1.55 | <0.0001 | 0.96 | 0.81 | 1.14 | 0.6790 |
| Second Degree Relative | 1.27 | 1.22 | 1.31 | <0.0001 | 1.01 | 0.89 | 1.16 | 0.8312 |
| First and Second Degree Relative | 1.58 | 1.51 | 1.65 | <0.0001 | 1.07 | 0.88 | 1.29 | 0.4980 |
| Don't Know | 1.21 | 1.14 | 1.28 | <0.0001 | 0.81 | 0.65 | 1.00 | 0.0544 |
| No Response/Missing | 1.29 | 1.14 | 1.45 | <0.0001 | 0.88 | 0.54 | 1.46 | 0.6281 |
| **Any Atypia Biopsies**^†^ |  |  |  |  |  |  |  |  |
| Not Biopsied | ref | ref | ref | ref | ref | ref | ref | ref |
| Biopsied - No ADH | 1.09 | 1.05 | 1.13 | <0.0001 | 1.09 | 0.95 | 1.24 | 0.2089 |
| Biopsied - Yes ADH | 1.46 | 1.32 | 1.61 | <0.0001 | 1.32 | 0.78 | 2.23 | 0.2951 |
| Biopsied - Don't know ADH | 1.38 | 1.25 | 1.52 | <0.0001 | 1.14 | 0.70 | 1.86 | 0.5969 |
| **Hormone Replacement Therapy Use Ever**^†^ | | | | | | | | |
| None | ref | ref | ref | ref | ref | ref | ref | ref |
| One or More | 1.09 | 1.05 | 1.12 | <0.0001 | 1.12 | 1.00 | 1.26 | 0.0511 |
| **Age (years) at Menarche**^†^ | | | | | | | | |
| 12 to 13 | ref | ref | ref | ref | ref | ref | ref | ref |
| Under age 12 | 0.96 | 0.93 | 1.00 | 0.0524 | 0.84 | 0.73 | 0.97 | 0.0174 |
| 14 or above | 1.01 | 0.98 | 1.05 | 0.4902 | 1.03 | 0.91 | 1.17 | 0.6064 |
| **Age (years) at first live birth**^†^ | | | | | | | | |
| Nulliparous | ref | ref | ref | ref | ref | ref | ref | ref |
| <20 years | 0.94 | 0.88 | 1.00 | 0.0428 | 0.91 | 0.72 | 1.15 | 0.4171 |
| 20-24 years | 0.95 | 0.91 | 1.00 | 0.0435 | 1.04 | 0.88 | 1.23 | 0.6237 |
| 25-29 years | 0.98 | 0.94 | 1.02 | 0.3736 | 1.00 | 0.86 | 1.17 | 0.9686 |
| 30-34 years | 1.04 | 0.99 | 1.08 | 0.1041 | 1.00 | 0.85 | 1.19 | 0.9606 |
| >34 years | 1.06 | 1.01 | 1.11 | 0.0166 | 0.98 | 0.81 | 1.19 | 0.8557 |
| **Baseline Area Deprivation Index^1^ (ADI)** | | | | | | | | |
| Least Disadvantaged (1 to 3) | ref | ref | ref | ref | ref | ref | ref | ref |
| Middle Disadvantaged (4 to 6) | 1.00 | 0.97 | 1.04 | 0.9654 | 0.98 | 0.86 | 1.11 | 0.7148 |
| Most Disadvantaged (7 to 10) | 0.95 | 0.91 | 0.99 | 0.0091 | 1.02 | 0.88 | 1.18 | 0.7951 |
| Data suppressed/ Not geocoded | 1.04 | 0.96 | 1.13 | 0.3284 | 1.24 | 0.94 | 1.63 | 0.1276 |

* Wald type III

^†^ denotes imputed variable

^1^ Kind & Buckingham, N. Engl. J. Med. 378, 2456–2458 (2018).

## Supplementary Table S4. Risk assessment and screening recommendations

| Risk: | Highest Risk | Elevated Risk | Average Risk | Lowest Risk |
| --- | --- | --- | --- | --- |
| Criteria/ Threshold | BRCA1/2, TP53, PTEN, STK11, CDH1 mutation carrier  OR  ATM, PALB2 or CHEK2 mutation carrier with positive family history of breast cancer  OR  Women with a ≥6% 5-year risk (risk of an average BRCA carrier)  OR  Women with a history of mantle radiation between ages 10-30 years | Women aged 40-49 with extremely dense breasts  OR  Women at a ≥1% 5-year risk of developing ER- breast cancer based on susceptibility SNPs  OR  Women in top 2.5^th^ percentile of risk by 1-year age category  OR  ATM, PALB2 or CHEK2 mutation carrier without a positive family history^**^ of breast cancer | Women aged 50-74  OR  Women aged 40-49 with a ≥1.3% 5-year risk (risk of an average 50 year-old woman) | Women aged 40-49 with a <1.3% 5-year risk of developing breast cancer  OR  Low risk (5-year risk <1.3%) and age ≥75  OR  Age ≥70, 5-year risk ≤2.2% and co-morbidities with predicted ≥50% risk of mortality in 10 years |
| Screening Rec: | Annual mammogram  Adjunct MRI  BHS active outreach  Risk reduction consultation | Annual mammogram*  BHS active outreach  Risk reduction consultation | Biennial mammogram† | No screening until age 50  OR  Stop screening |

^*^ If individual does not meet criteria for annual mammogram + MRI

^†^ If individual does not meet criteria for annual mammogram or annual mammogram + MRI

^**^ Family history is defined as a first degree relative with breast cancer, two second-degree relatives with breast cancer, or one second-degree relative diagnosed prior to age 45.

*(previously published in Esserman et al. NPJ Breast Cancer. 2017;3(1):34. doi:10.1038/s41523-017-0035-5)*

**Supplementary Table S5:** WISDOM Study and Athena Breast Health Network Consortia Members

**University of California, San Francisco**

| Advika Verma | Jennie Ling | Olivia Cooper |
| --- | --- | --- |
| Alexandra Solomon | Jennifer Atamer | Paige Warner |
| Allison Stover Fiscalini | Jennifer James, PhD | Patricia Choy |
| Alondra Torres | Jenny Domnich | Pamela Williams |
| Amie Blanco, CGC | Julissa Cabrera | Patrick Shibley |
| Barbara Koenig, PhD | Katherine Leggat-Barr | Rashna Soonavala |
| Barry Tong, CGC | Katherine Ross, CGC | Rita Freimanis, MD |
| Celia Kaplan, PhD | Kaylena Principe | Robert Hiatt, PhD |
| Chomba Muwele | Kelly Adduci | Roxanna Firouzian |
| Christina Yau, PhD | Kelly Blum | Sara Nelson |
| Daisy Montoya | Kenneth Wimmer | Sarah Knopf |
| Dan Dumitru | Kimberly Badal, PhD | Sarah Rosenberg-Wohl |
| Donglei Hu | Kirkpatrick Fergus, MD | Sarah Theiner |
| Dylan Dumitru | Laura Esserman, MD, MBA | Scott Huntsman |
| Elad Ziv, MD | Laura van ‘t Veer, PhD | Setareh Sarrafan |
| Elene Tsopurashvili | Leah Sabacan | Shreya Kapoor |
| Emily Smith | Madeline Matthys | Steffanie Goodman |
| Galen Joseph, PhD | Mandy Che | Stephanie Flores |
| Halle Thannickal | Marcelo Palmieri | Suzie Eder |
| Heather Greenwood, MD | Maren Scheuner Purcell, MD MPH | Taylor Glatt |
| Hillary Hsu | Maydha Dhanuka | Tianyi Wang |
| Holly Keane, MD | Michael Resendez | Tomiyuri Lewis |
| Irene Acerbi Soto, PhD | Mi-Ok Kim, PhD | Yash Huilgol |
| Jackelyn Moya, MD | Nandini Seth | Yisell Morera |
| Jeff Tice, MD | Natalie Kim | Yunibia Lopez |

**University of California, Los Angeles**

| Alyssa Rocha, BA | Carlie Thompson, MD | Neil Wenger, MD |
| --- | --- | --- |
| Anne Hoyt, MD | Erica Silver, MS | Sitaram Vangala, MS |
| Annette Stanton, PsyD | Guita Rahbar, MD | Xochil Calderon, MPH |
| Antonia Petruse, MBA | Jacqueline Trent |  |
| Arash Naeim, MD, PhD | Liliana Johansen, BA |  |

**University of California, Davis**

| Alexander Borowsky, MD | Josh Fenton, MD | Samrrah Raouf, PhD, CCRP |
| --- | --- | --- |
| Diana Miglioretti, PhD | Lydia Howell, MD | Skye Stewart, MPH |
| Elizabeth Morris, MD | Richard Bold, MD |  |

**WISDOM Study and Athena Breast Health Network Consortia Members**, cont.

**University of California, Irvine**

| Andrea Alvarez | Hannah Lui Park, PhD | Lorin Kirmizi, BS |
| --- | --- | --- |
| Argyrios Ziogas, PhD | Hannah Schluter, BS | Melodie Elyasian, BS |
| Caressa Wong, BS | Hoda Anton-Culver, PhD | Richard Kelly, BS |
| Christie Lee, BS | Jasmin Hassam, BS | Simar Salopal, BS |
| Danielle Forman, BS | Karen Lane, MD | Stephen Feig, MD |
| Deborah Goodman, MD, PhD | Kari Kansal, MD |  |
| Freddie Combs, MD | Kathy Larsen, MD |  |

**University of California, San Diego**

| Andrea LaCroix, PhD | John Pierce, PhD | Richard Schwab, MD |
| --- | --- | --- |
| Anne Wallace, MD | Karen Messer, PhD | Sara Fainstein, PhD |
| Ava Hosseini, MD | Katie Abihider | Sarah Blair, MD |
| Barbara Parker, MD | Kimberly Forbes, MS | Sheri Hartman, PhD |
| Diana DeRosa, CGC | Lisa Madlensky, PhD | Steele Fors |
| Farnaz Hasteh, MD | Mary Devereaux, MD | Tracy Layton, MS |
| Haydee Ojeda-Fournier, MD | Melanie Fiorella, MD | Vedeline Torreon |
| John Einck, MD | Michael Hogarth, MD |  |

**Sanford Health**

| Andrea Kaster, MD | Jesse Dirksen, MD | Sharon Hunt |
| --- | --- | --- |
| David Pearce | Kathy Hanish | Thomas Cink, MD |
| Heather Harvey, BSN, RN | Larissa Risty, MS, CGC |  |
| Jan Wernisch, BSN | Nancy Anderson |  |

**University of Chicago**

| Abigail Yuhan | Erin Kim | Jan-Marie Mc Evilly |
| --- | --- | --- |
| Anna Woodard | Hatice Basdag | Naoman Arif, CRC |
| Arnaaz Khwaja | Hiroyuki Abe, MD | Olufunmilayo Olopade, MD |
| Brenda Gonzales, CRC | Ian Hurley | Sebastian Perez |
| Cara Essling | Ilona Siljander | Thomas Li |
| Dezheng Huo, PhD | Iris Romero, MD | Wenji Guo, MD |

**University of Alabama – Birmingham**

| Rachael Lancaster, MD | Stacey Ingram, Med, CHES | Tara Bowman, MSW |
| --- | --- | --- |
| Le’Andrea Anderson, BS |  |  |

**Louisiana State University**

| Agustin Garcia, MD | Eileen Mederos, RN | Peyton Percle, MA |
| --- | --- | --- |
| Brooke Stewart, MPH | Laura Siqueros |  |

**Diagnostic Center of Miami**

| James Esserman, MD | Isabella Cabaleiro, MS | Michael Plaza, MD |
| --- | --- | --- |

**WISDOM Study and Athena Breast Health Network Consortia Members**, cont.

**San Francisco VA Health Care System**

| Paloma Sales, PhD | Julia Cover | Tessa Miller |
| --- | --- | --- |

**DHR Health**

| Angela Varela Espinosa | Lisa Trevino | Vaney Capetillo |
| --- | --- | --- |
| Lisa Chapa, MD | Sohail Rao, MD |  |

| **Weill Cornell Medicine**  Yiwey Shieh, MD | **Massachusetts General**  Karen Sepucha, PhD |
| --- | --- |
|  |  |
| **Karolinska Institutet**  Martin Eklund, PhD | **Virginia Commonwealth University**  Kim Rhoads, MD, MS, MPH |
|  |  |
| **Kaiser Permanente Division of Research**  Vignesh Arasu, MD, PhD |  |

**WISDOM Community Leadership Board**

| Dolores Moorehead, MS, APCC | Lisa Tealer, BS | Olivia Fe |
| --- | --- | --- |
| Debra Oto-Kent, MPH | Marion Harris, RN | Tau Ve’e, MBA |
| Dhalia Balmir | Nia Bailey | Wilma Batiste, CRC, NCPT |

**WISDOM Patient Advocates**

| Diane Heditsian, BA | Michele Rakoff | Susie Brain, BSc |
| --- | --- | --- |
| Dolores Morehead, MS, APCC | Patricia Haugen | Vivian Lee, MS |
| Jo Ellen Lezotte | Sandy Finestone, PsyD |  |
